# Adaptation of *Bacillus subtilis* to Life at Extreme Potassium Limitation

**DOI:** 10.1128/mBio.00861-17

**Published:** 2017-07-05

**Authors:** Jan Gundlach, Christina Herzberg, Dietrich Hertel, Andrea Thürmer, Rolf Daniel, Hannes Link, Jörg Stülke

**Affiliations:** a Department of General Microbiology, Georg-August University Göttingen, Göttingen, Germany; b Department of Plant Ecology and Ecosystems Research, Georg-August University, Albrecht-von-Haller-Institute, Göttingen, Germany; c Department of Genomic and Applied Microbiology, Georg-August University Göttingen, Göttingen, Germany; d Max Planck Institute for Terrestrial Microbiology, Dynamic Control of Metabolic Networks, Marburg, Germany; Duke University School of Medicine

**Keywords:** *Bacillus subtilis*, arginine biosynthesis, c-di-AMP, ion homeostasis, potassium transport

## Abstract

Potassium is the most abundant metal ion in every living cell. This ion is essential due to its requirement for the activity of the ribosome and many enzymes but also because of its role in buffering the negative charge of nucleic acids. As the external concentrations of potassium are usually low, efficient uptake and intracellular enrichment of the ion is necessary. The Gram-positive bacterium *Bacillus subtilis* possesses three transporters for potassium, KtrAB, KtrCD, and the recently discovered KimA. In the absence of the high-affinity transporters KtrAB and KimA, the bacteria were unable to grow at low potassium concentrations. However, we observed the appearance of suppressor mutants that were able to overcome the potassium limitation. All these suppressor mutations affected amino acid metabolism, particularly arginine biosynthesis. In the mutants, the intracellular levels of ornithine, citrulline, and arginine were strongly increased, suggesting that these amino acids can partially substitute for potassium. This was confirmed by the observation that the supplementation with positively charged amino acids allows growth of *B. subtilis* even at the extreme potassium limitation that the bacteria experience if no potassium is added to the medium. In addition, a second class of suppressor mutations allowed growth at extreme potassium limitation. These mutations result in increased expression of KtrAB, the potassium transporter with the highest affinity and therefore allow the acquisition and accumulation of the smallest amounts of potassium ions from the environment.

## IMPORTANCE

Potassium is essential for every living cell as it is required for the activity for many enzymes and for maintaining the intracellular pH by buffering the negative charge of the nucleic acids. We have studied the adaptation of the soil bacterium *Bacillus subtilis* to life at low potassium concentrations. If the major high-affinity transporters are missing, the bacteria are unable to grow unless they acquire mutations that result in the accumulation of positively charged amino acids such as ornithine, citrulline, and arginine. Supplementation of the medium with these amino acids rescued growth even in the absence of externally added potassium. Moreover, these growth conditions, which the bacteria experience as an extreme potassium limitation, can be overcome by the acquisition of mutations that result in increased expression of the high-affinity potassium transporter KtrAB. Our results indicate that positively charged amino acids can partially take over the function of potassium.

## INTRODUCTION

In each living cell, potassium and glutamate are by far the most abundant cation and anion, respectively. *Escherichia coli* accumulates the two ions to intracellular concentrations of between 200 and 400 mM for potassium and about 100 mM for glutamate (1, 2). With this concentration, glutamate alone accounts for about one-third of the cellular metabolite pool in *E. coli* and other bacteria (3). Potassium acts not only as a counterion for glutamate, but it is also required for maintaining the intracellular pH by buffering the negative charge of nucleic acids. Moreover, potassium is required for the control of gene expression, activation of enzymes, and adaptation to osmotic stress, and it is essential for ribosome function (1, 4–7).

**FIG 1:**
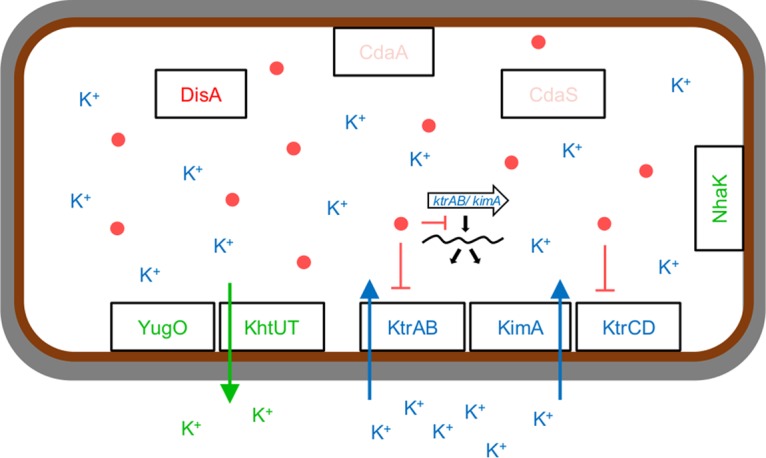
Cyclic di-AMP controls K^+^ uptake. When the external K^+^ concentration is low, c-di-AMP synthesis is reduced. The high-affinity K^+^ transporters *ktrAB* and *kimA* are expressed and c-di-AMP is transported into the cell. When the external K^+^ concentrations are high, c-di-AMP synthesis is increased. The second messenger binds to the *ktrAB* and *kimA* riboswitch (indicated by the large white arrow), preventing transcription of the respective genes encoding the high-affinity transporters. In addition, c-di-AMP negatively controls the activity of KtrAB and KtrCD by binding to the regulatory components KtrA and KtrC, respectively. YugO and KhtUT are K^+^ exporters, and NhaK is a monovalent cation/H^+^ antiporter (10). Red dots, c-di-AMP molecules.

While nearly all bacteria possess pathways for glutamate biosynthesis, the essential metal ion potassium can be acquired only by transport. Given potassium concentrations of only 100 µM to 10 mM in typical bacterial habitats, highly effective uptake systems are required to accumulate and enrich potassium up to 2,000-fold (8). Most bacteria possess multiple high- and low-affinity potassium uptake systems to allow efficient transport under all environmental conditions. In the Gram-positive model organism *Bacillus subtilis*, three different potassium transporters have been identified. KtrAB and KtrCD are hetero-oligomeric transporters that consist of a membrane-spanning channel (KtrB and KtrD) and a peripheral regulatory subunit (KtrA and KtrC) (9). Moreover, we have recently discovered KimA, the founding member of a novel widespread family of potassium transporters in bacteria (10). While KtrCD is a low-affinity transporter that is important for potassium uptake under most environmental conditions, KtrAB and KimA are high-affinity transporters that allow rapid growth at micromolar potassium concentrations (10). In good agreement, the *ktrC* and *ktrD* genes are constitutively expressed, whereas expression of the *ktrAB* operon and the *kimA* gene is very low (11). The *ktrAB* and *kimA* transcription units are under the control of a riboswitch that responds to the essential second messenger cyclic di-AMP (c-di-AMP) (12). Transcription beyond this riboswitch is possible only at low intracellular c-di-AMP concentrations (10, 12). Another level of control of potassium uptake is exerted via the regulatory domains of KtrAB and KtrCD: the regulatory subunits contain so-called RCK_C domains (regulator of conductance of K^+^). These domains bind the second messenger c-di-AMP, resulting in inactivation of the transporters (13). Thus, high levels of c-di-AMP prevent both expression of the high-affinity potassium transporters and the activity of the KtrAB and KtrCD uptake systems (14) (Fig. 1).

Potassium homeostasis is controlled not only by its uptake but also by export proteins. In *B. subtilis*, the potassium/proton antiporter KhtTU has been functionally characterized (15, 16). Moreover, the YugO protein is a glutamate-responsive potassium channel that has also been implicated in cell-to-cell communication in *B. subtilis* biofilms (17, 18).

We are interested in signal transduction in the model bacterium *B. subtilis* by the second messenger c-di-AMP. This signaling nucleotide is unique in two respects. First, c-di-AMP is the only known essential second messenger; *B. subtilis* and related bacteria such as *Staphylococcus aureus* or *Listeria monocytogenes* that produce this molecule are not viable in its absence (19–22). Importantly, c-di-AMP is not only essential but also toxic if it accumulates to high concentrations (23). Second, c-di-AMP is the only second messenger that controls a biological process by binding to a protein (KtrA) and to the corresponding mRNA molecule, the riboswitch that controls *ktrAB* operon expression. The reason for the essentiality of c-di-AMP has long remained enigmatic. Among all known target molecules in *B. subtilis*, the potassium transporter subunit KtrA, the signal transduction protein DarA, and the *kimA* riboswitch, none has been implicated in essentiality and toxicity of c-di-AMP (12, 13, 23, 24). Recently, we have shown that potassium homeostasis is a complex process that is causal for the essentiality of c-di-AMP: in the absence of c-di-AMP, potassium becomes toxic at low concentrations, whereas an excess of the second messenger is likely to result in an inability to acquire the essential potassium ions. Indeed, c-di-AMP is dispensable if the cells are cultivated at low external potassium concentrations (10).

In this study, we have analyzed the response of *B. subtilis* to potassium limitation. The characterization of suppressor mutants revealed that positively charged amino acids can partially substitute for potassium. However, potassium remains essential for the bacteria, and the cells respond to extreme potassium limitation with mutations that allow increased expression of the high-affinity uptake system KtrAB. These mechanisms allow the bacteria to cope with potassium limitation and to acquire and enrich even the smallest amounts of potassium ions from their environment.

## RESULTS

### Suppressor mutations in strains lacking high-affinity potassium transporters.

**FIG 2:**
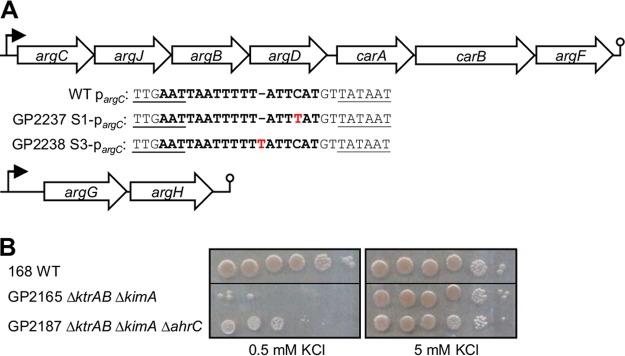
Suppressor mutations allowing growth of a *ktrAB kimA* mutant at low potassium concentrations. (A) Genetic organization of the *argCJBD-carAB-argF* and *argGH* operons. Wild-type (WT) and mutant *argC* promoter sequences (strains GP2237 and GP2239) are shown. Conserved regions are underlined. The AhrC binding site is highlighted. Point mutations are indicated in red. (B) Growth assay of the *ktrAB kimA* mutant in the presence (GP2165) and absence (GP2187) of AhrC at low (0.5 mM) and high (5 mM) potassium concentrations (*n* = 3). The black lines indicate that different areas of the same plates that have been joined together are shown in the images shown in this panel.

We have recently identified the novel high-affinity potassium transporter KimA (10). Simultaneous inactivation of both high-affinity uptake systems, KimA and KtrAB, prevented growth at low potassium concentrations; however, we observed the rapid appearance of suppressor mutants (10). We hypothesized that such suppressor mutations might affect other ion uptake systems to allow potassium transport with high affinity. To identify the mutations responsible for the suppression, we determined the genome sequence of three independent mutants for the *kimA ktrAB* background. Surprisingly, none of the mutations affected any ion transporters. We observed single point mutations in all three strains. Two of these mutations affected the upstream region of the *argCJBD-carAB-argF* operon (Fig. 2A), and the third mutation affected the *ahrC* repressor gene and resulted in a single amino acid substitution (Q22R). As shown in Fig. 2A, the point mutations in the *argC* promoter region directly affect the AhrC binding site (25, 26), suggesting that altered control of the *argC* operon by AhrC is responsible for the suppressive effect of the mutations. To test this hypothesis, we deleted the *ahrC* gene in the background of the *kimA ktrAB* mutant GP2165 and analyzed growth of the resulting strain, GP2187, at low potassium concentration (0.5 mM KCl). Indeed, the deletion of *ahrC* was sufficient to restore growth of the *kimA ktrAB* mutant (Fig. 2B). Thus, loss of arginine biosynthetic operon repression by AhrC is sufficient for the *kimA ktrAB* mutant to overcome potassium limitation.

We also tested other strains with mutations affecting potassium transporters. The *kimA ktrC* (GP2166) double mutant and the *ktrAB yugO* (GP2170) double mutant also acquired suppressor mutations. Whole-genome sequencing of these mutants identified a frameshift mutation in *odhA* encoding the E1 subunit of the 2-oxoglutarate dehydrogenase and a large genomic deletion (about 200 kb; from *yobH* to *uvrX*), respectively. Importantly, the *yobH* and *uvrX* genes share a stretch of 641 bp with 92% identical nucleotides, suggesting that the deletion occurred by homologous recombination. Strikingly, this deletion occurred between prophage 6 and the SPβ region. This region also encompasses the *odhAB* operon for the 2-oxoglutarate dehydrogenase. In one additional *kimA ktrAB* mutant, we did not observe a suppressor mutant affecting control of arginine biosynthesis as described above. Instead, sequencing of the *odhA* gene of this suppressor strain (GP2703) identified point mutations in the *odhA* gene, which result in amino acid substitutions of Arg-336 and Ala-337 to Glu and Pro, respectively. Due to the poor growth of *odhA* mutants, we did not further analyze these mutations.

### Positively charged amino acids can partially substitute potassium.

**FIG 3:**
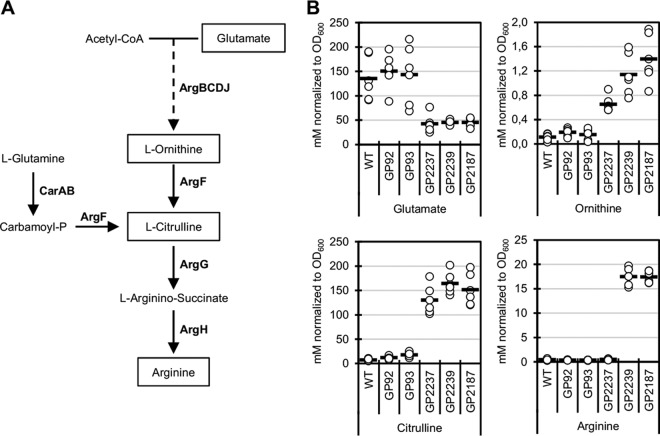
The suppressor mutations result in accumulation of amino acids of the glutamate family. (A) Arginine biosynthetic pathway. Acetyl-CoA, acetyl coenzyme A. (B) Intracellular levels of glutamate, ornithine, citrulline, and arginine in wild-type (WT) cells, single transporter mutants (*ktrAB* [strain GP92] and *kimA* [GP93] mutants), and in suppressor mutants affecting the AhrC binding site (strain GP2237) and AhrC (GP2239) as well as in the *ktrAB kimA ahrC* mutant strain (GP2187). All strains were grown in MSSM medium with 0.5 mM KCl. Each circle represents the value for a biologically independent replicate, and the short horizontal bars indicate the mean values of the replicates (*n* = 6).

The isolation of mutations that are likely to affect the arginine biosynthetic pathway (Fig. 3A) and arginine accumulation by redirecting metabolism at the 2-oxoglutarate node to the arginine precursor glutamate suggested that alterations in amino acid metabolism cause suppression of the potassium transporter mutants. To address this question, we determined the intracellular concentrations of glutamate, ornithine, citrulline, and arginine in the wild-type *B. subtilis* strain 168, single *ktrAB* and *kimA* mutants GP92 and GP93, and suppressor strains with mutations in the *ahrC* binding site of the *argC* promoter region (GP2237) and in the *ahrC* gene (GP2239). Finally, we investigated the *ktrAB kimA* mutant with the *ahrC* gene deleted (GP2187). As shown in Fig. 3B, the levels of glutamate were high in the wild type and in the single mutants but low in both suppressor mutants and in the *ahrC* deletion mutant. In contrast, the intracellular ornithine and citrulline concentrations were increased in the latter three strains, suggesting an upregulation of the upper arginine biosynthesis pathway. The arginine concentrations were very low in the wild type, the transporter single mutant strains, and the strain with the mutation in the *ahrC* binding site of the *argC* promoter (GP2237). However, the arginine concentrations were substantially increased in both *ahrC* mutants (from 0.4 to about 18 mM normalized to growth at an optical density at 600 nm [OD_600_]). The differential effect of the suppressor mutations on the ornithine, citrulline, and arginine concentrations is in excellent agreement with the arginine biosynthetic pathway and its regulation. All genes required for ornithine and citrulline biosynthesis from glutamate are contained in the *argCJBD-carAB-argF* operon which is derepressed due to the mutation in the AhrC binding site (26). Thus, this single point mutation is sufficient for increased ornithine and citrulline synthesis. In contrast, the further pathway from citrulline to arginine requires the enzymes encoded by a second operon, *argGH* (Fig. 2A). This operon is also subject to repression by AhrC (27). Accordingly, increased arginine biosynthesis is possible only if both operons are derepressed due to mutations in *ahrC*.

**FIG 4:**
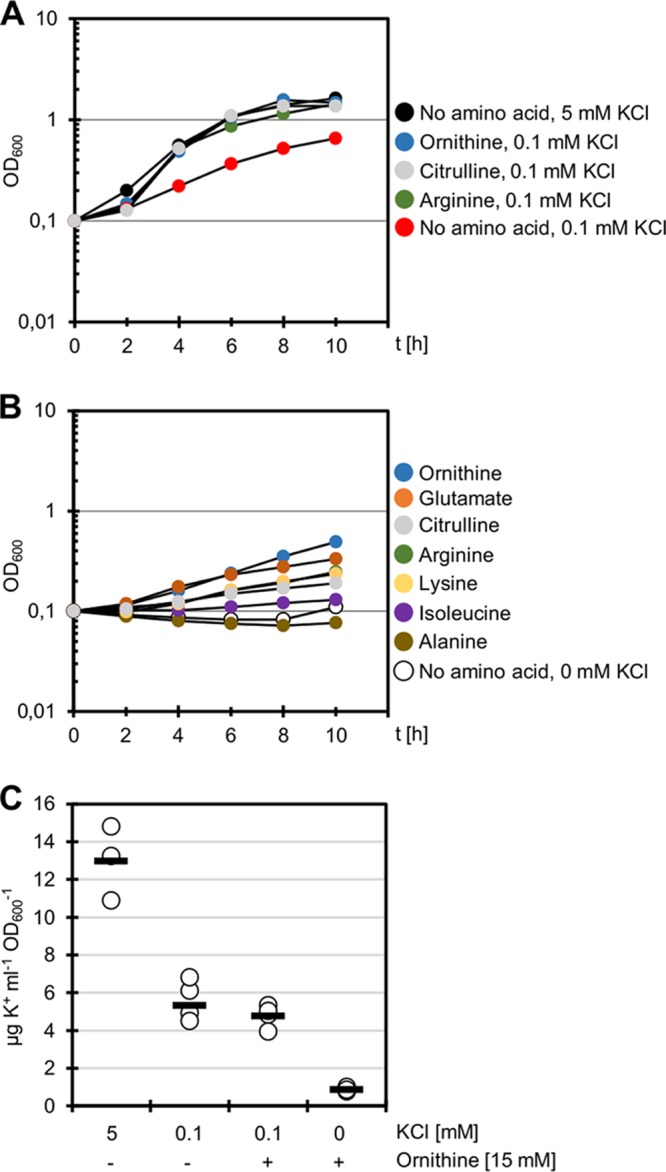
Positively charged amino acids can partially compensate for the lack of potassium in the medium. (A) Growth assay of *B. subtilis* 168 in the presence of 0.1 mM KCl and 0.1 mM KCl with the addition of ornithine, citrulline, and arginine (15 mM each). The *y* axis shows time (t) (in hours). As a control, the wild type was grown in the presence of 5 mM KCl (black) (*n* = 3). (B) Growth assay of *B. subtilis* 168 in the absence of potassium (white) and in the absence of potassium with the addition of ornithine, glutamate, citrulline, arginine, lysine, isoleucine, and alanine (15 mM each) (*n* = 3). (C) *B. subtilis* 168 was grown at 5 mM KCl, 0.1 mM KCl with and without the addition of ornithine (15 mM), and without external potassium with the addition of ornithine (15 mM). The cellular K^+^ concentrations were determined by ICP-OES. Circles represent the values for biologically independent replicates, and the short horizontal bars indicate the mean values of the replicates (*n* = 3 for 5 mM KCl; *n* = 6 for all other conditions).

The suppressor mutations affecting the arginine biosynthetic operons resulted in higher intracellular concentrations of ornithine, and they restored growth at low potassium concentrations in a strain lacking the two high-affinity potassium transporters. Thus, we asked whether there is a causal relation between ornithine/citrulline accumulation and growth at low potassium concentrations. To address this question, we compared the growth of the wild-type *B. subtilis* strain 168 at low (0.1 mM) and high (5 mM) potassium concentrations in the medium. As shown in Fig. 4A, growth was possible but significantly delayed at 0.1 mM potassium. Next, we tested the effect of adding ornithine, citrulline, or arginine to the growth medium (Fig. 4A). All three amino acids resulted in improved growth, as with high potassium concentrations. These results suggest that these amino acids can at least partially replace the cellular function of potassium. To explore this hypothesis further, we cultivated *B. subtilis* 168 in MSSM minimal medium without adding any potassium ions. Under this condition, no growth was possible (Fig. 4B). However, the addition of ornithine, citrulline, or arginine rescued growth even in the absence of externally added potassium.

The results described above raised two immediate further questions. (i) Were the bacteria able to grow without any potassium as long as they were provided with these amino acids? (ii) Is this substitutive effect limited to ornithine, citrulline, and arginine, or are other amino acids also capable of substituting for potassium? To answer the first question, we determined the intracellular potassium concentrations of *B. subtilis* 168 during growth with 5 mM and 0.1 mM potassium. Moreover, potassium concentrations were assayed for cells cultivated with 0.1 mM potassium or without potassium in the presence of ornithine (15 mM) (Fig. 4C). As observed previously, the potassium levels were highest in cells grown with 5 mM external potassium. At 0.1 mM potassium, low cellular potassium levels were determined irrespective of the presence of ornithine in the medium. When the bacteria were cultivated in the absence of externally added potassium but in the presence of ornithine as substituent, potassium was still detected in the cells but at very small amounts (Fig. 4C). Thus, potassium is available in the medium without external supply, likely due to the presence of potassium ions in other chemicals or to potassium release from the glassware. Our results suggest that the cells are capable of transporting potassium into the cell even at extremely low concentrations. However, we conclude that the bacteria suffer from an extreme potassium limitation at these concentrations.

To analyze the potential of other amino acids to substitute for potassium, we grew *B. subtilis* 168 in MSSM medium without added potassium. In addition to ornithine, citrulline, and arginine, we tested the effect of glutamate, the precursor of these amino acids. Moreover, we used lysine, alanine, and isoleucine (Fig. 4B). The best growth was observed in the presence of the positively charged amino acids ornithine, citrulline, arginine, and lysine as well as with glutamate, the precursor for the biosynthesis of ornithine, citrulline, and arginine. In contrast, no growth was possible if alanine or isoleucine were added to the medium. We conclude that only the positively charged amino acids (and their precursor glutamate) can partially substitute for potassium.

### Increased expression of KtrAB shifts the minimal potassium requirements.

**FIG 5:**
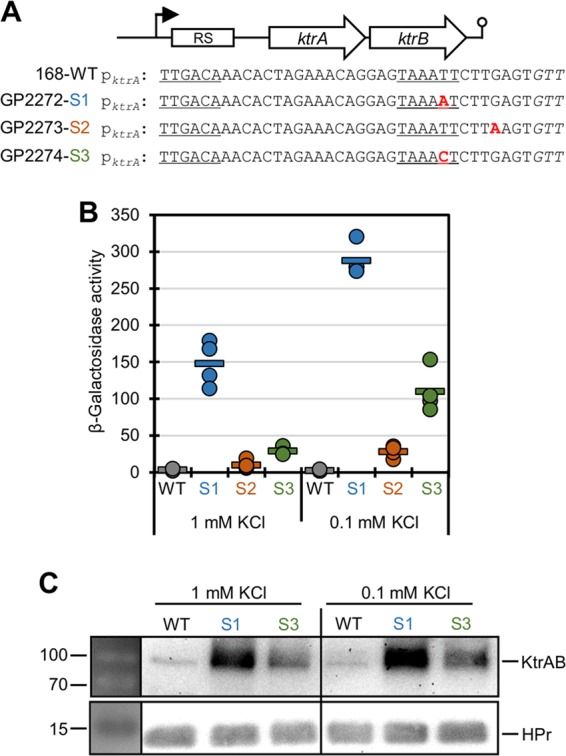
Expression of the *ktrAB* operon. (A) Sequence alignment of the *ktrAB* promoter of the wild-type (WT) strain and three suppressor mutants (S1 to S3 mutations in strains GP2272 to GP2274, respectively). Conserved regions are underlined, and point mutations are highlighted in red. (B) The wild-type *ktrA* promoter region (strain GP2299) and the promoter regions of three *ktrA* promoter mutants (S1 to S3 mutations in strains GP2300, GP2714, and GP2701, respectively) were fused to *lacZ*. Bacterial cells were grown at the indicated potassium concentrations, and promoter activities were determined by quantification of β-galactosidase activities. Each circle represents the value for an individual biological replicate. The short horizontal bar represents the mean value (*n* = 4). (C) Determination of intracellular levels of KtrB labeled with a triple FLAG tag in the wild type (strain GP2277) and two different *ktrAB* promoter mutants (S1 and S3 mutations in GP2278 and GP2280 strains, respectively). Bacterial cells were grown at the indicated potassium concentrations, and KtrB was detected using antibodies recognizing the FLAG tag. The HPr protein served as the control (*n* = 3). The molecular weights (in kilodaltons) are shown to the left of the gels.

As described above, *B. subtilis* 168 was unable to grow in the absence of externally provided potassium ions unless amino acids of the glutamate family were added. However, cells grew after overnight incubation at extreme potassium limitation. This observation suggested the acquisition of suppressor mutations that allowed growth. Indeed, upon reinoculation, the suppressor mutants were able to grow in medium without added potassium. Whole-genome sequencing of one suppressor mutant (GP2272) indicated a point mutation in the promoter region of the *ktrAB* operon. Moreover, we have determined sequences of the *ktrAB* promoter regions of 12 additional mutants. All of them carried single point mutations in this region (Fig. 5A). Of the 13 mutations, 12 affected a single position in the putative −10 region of the *ktrAB* operon promoter, the T at position −9 (T_−9_). This nucleotide was replaced by either A (T_−9_ A) or C (T_−9_ C). Representative strains for the two mutations are GP2272 and GP2274, respectively. In one mutant (GP2273), there was a single point mutation at position −4 which resulted in a G-to-A substitution (Fig. 5A).

The presence of point mutations in the *ktrAB* promoter region in many independently isolated suppressor mutants suggested that these mutations might result in increased *ktrAB* expression and subsequently in more efficient uptake of trace amounts of potassium. To test this hypothesis, we constructed reporter fusions of the *ktrAB* promoter region to a promoterless *lacZ* gene encoding β-galactosidase. Moreover, we constructed strains expressing KtrB with a C-terminal FLAG tag to facilitate protein detection by Western blot analysis. For the wild-type *ktrAB-lacZ* fusion present in strain GP2299, no expression was detected even at a low potassium concentration (0.1 mM). In contrast, the promoter point mutation S1 (T_−9_ A; strain GP2300) rendered the promoter active at both potassium concentrations (1 mM and 0.1 mM) tested in this study. The mutation S2 at position −4 (strain GP2714) resulted in slightly increased reporter activity, particularly at the lower potassium concentration. The promoter point mutation S3 (T_−9_ C; strain GP2701) allowed an intermediate promoter activity which was still responsive to the potassium concentration (Fig. 5B). In agreement with the reporter assay results, the intracellular amounts of KtrB, the product of the second gene of the *ktrAB* operon, were increased as a result of the promoter point mutations at the position T_−9_ (mutations S1 and S3) compared to the wild-type strain (Fig. 5C).

To test whether the most effective promoter mutation (S1) also affects potassium accumulation, we determined the cellular potassium levels of the wild-type strain 168 and the suppressor mutant GP2272. The results for the wild-type strain are shown in Fig. 4C. For the *ktrAB* promoter mutant, very similar results were obtained. At 0.1 mM external potassium, the wild type and the mutant strains had accumulated 5.35 and 5.27 µg K^+^ ml^−1^ OD_600_ unit^−1^, respectively. If the cells were grown with ornithine but without externally added potassium, 0.88 and 0.99 µg K^+^ ml^−1^ OD_600_ unit^−1^, respectively, were determined for the two strains. Thus, the increased amounts of KtrAB do not directly result in increased intracellular potassium accumulation. Unfortunately, we were unable to compare the potassium levels of the two strains in the absence of both potassium and ornithine due to the inability of the wild-type strain to grow under these conditions.

## DISCUSSION

Potassium is a macroelement that is essential for any living cell. Accordingly, organisms have evolved low- and high-affinity potassium uptake systems that allow the efficient acquisition of this ion in a wide range of environmental conditions. In this study, we have analyzed the adaptation of *B. subtilis* to potassium limitations caused by (i) lack of the high-affinity potassium transporters and (ii) lack of external potassium supply. Our results indicate that the bacteria are able to partially substitute for potassium by synthesizing positively charged amino acids of the glutamate family. This is possible if some potassium can still be transported due to the presence of the low-affinity uptake system KtrCD. Even under conditions of extreme potassium limitation, the availability of amino acids of the glutamate family supports growth. However, in the absence of these amino acids, the cells have the ability to adapt rapidly by acquiring mutations that increase the expression of the high-affinity transporter KtrAB.

The observation that loss of the high-affinity potassium transporters KtrAB and KimA resulted in the appearance of suppressor mutants immediately suggested that these mutants had acquired novel high-affinity potassium transporters, probably by extending the specificity of other metal ion transporters. This is not unprecedented as we have observed that mutated variants of the NhaK cation exporter can achieve increased activity toward potassium (10). However, none of the mutants that we have analyzed carried mutations affecting any gene encoding a potassium transporter. Surprisingly, all mutations affected amino acid metabolism either by redirecting the carbon metabolism from the citric acid cycle toward glutamate or by altering the regulation of the arginine biosynthetic genes. Similar results were obtained with suppressor mutants compensating for the lack of major magnesium transporters (28). Similar to potassium, magnesium is essential for bacteria (29). However, suppressor mutations that allow growth in the absence of magnesium transporters are not directly related to ion transport, indicating an indirect suppression mechanism (28).

The adaptation by the synthesis of positively charged amino acids has two distinct facets that reflect the different functions of potassium in the cell: the ion is absolutely essential for translation in the ribosome. Therefore, at least some potassium acquisition is required under all conditions. This residual potassium uptake is likely due to the activity of the remaining low-affinity transporter KtrCD. The suppressor mutants resulting from loss of the high-affinity transporters KtrAB and KimA were isolated at a potassium concentration of 0.5 mM. Under these conditions, KtrCD, although it has only low affinity for potassium (9, 10), may at least have some residual activity. Moreover, expression of KtrCD was shown to be increased upon loss of KtrAB and KimA (9). This residual uptake capacity provides the cell with the potassium required to meet the most urgent need of the ribosome. Indeed, the KtrCD system was essential in our *ktrAB kimA* double mutant strain, indicating that KtrCD is responsible for the residual potassium supply. However, the second function of potassium, buffering the negative charge of nucleic acids, likely requires higher intracellular potassium concentrations than those provided by KtrCD alone. The identification of suppressor mutations that relieve the repression of the arginine biosynthetic genes and thus the increased synthesis of ornithine, citrulline, and arginine suggests that these amino acids take over the buffering function. Among these three amino acids, citrulline reaches by far the highest concentrations in the suppressor mutants. This finding strongly suggests that citrulline replaces potassium in buffering the negative charge of the nucleic acids in the cell. It is interesting to note that growth at extremely low potassium concentration has also been observed for *Corynebacterium glutamicum* and *Bacillus stearothermophilus* (30, 31). For the latter bacterium, replacement of potassium by the addition of glutamate and ammonium was reported, and the substituting effect was ascribed to the ammonium ions (31). However, the work presented here suggests that the positively charged amino acids that can be produced if glutamate and ammonium are present in the medium partially take over the function of potassium.

Even in the absence of externally added potassium, the cells are capable of growing as long as they are provided with positively charged amino acids. However, determining the potassium pools of the cells revealed that the bacteria are still able to accumulate potassium even from this medium that contains potassium ions only due to impurities of other medium components. Moreover, in the absence of amino acids, suppressor strains with mutations affecting expression of the high-affinity potassium transporter KtrAB were isolated. Among 13 isolated mutants, all had point mutations in the *ktrAB* promoter region. These mutations affected either the −10 region of the promoter or the position −4 and resulted in increased *ktrAB* expression as determined by assays of the amounts of KtrB protein and studies of the promoter activity. Interestingly, we did not observe activity of the wild-type *ktrAB* promoter under the conditions applied in this study. It is likely that the operon is expressed only at extremely low potassium concentrations that limit growth of the bacteria. As observed for expression of the *kimA* gene encoding the second high-affinity potassium transporter (10), the control of *ktrAB* expression by potassium may be achieved not only by the c-di-AMP-responsive riboswitch but additionally by an as yet unknown transcription factor. Thus, we cannot exclude the possibility that our reporter constructs lack a binding site for an activator protein that binds further upstream of the promoter. Since all tested mutants contained mutations in the *ktrAB* promoter region, it is reasonable to assume that these mutations are the major way to achieve increased expression of the *ktrAB* operon. A transposon mutant screen for mutations resulting in deregulation of *kimA* and *ktrAB* identified *trans*-acting mutations in the *ndh* and *menH* genes (32). However, the mechanism by which these mutations that interfere with respiration and the citric acid cycle result in increased *kimA* and *ktrAB* expression has not yet been elucidated.

*B. subtilis* lives in soil and on plant surfaces. The bacteria can readily adapt to high and low external potassium concentrations by expressing the low- and high-affinity potassium transporters, respectively. However, during a heavy shower, the external potassium will be diluted to minute amounts. Under these conditions, the bacteria can still adapt by acquiring mutations that result in increased KtrAB expression, and thus still allow uptake of residual potassium amounts.

Metal ions are essential for many processes in the cell, but their accumulation can result in toxicity. Bacteria have therefore evolved processes to control the homeostasis of the different metal ions, including regulated uptake and efflux systems. The mechanisms that govern metal ion homeostasis have been extensively studied in *B. subtilis* for divalent cations (33). In contrast, little is known on the control of potassium homeostasis in *B. subtilis*. At high external potassium concentrations, the uptake of the ion is prevented by the essential second messenger c-di-AMP, thus preventing potassium intoxication. With the work presented in this study, we have extended the mechanisms implicated in controlling potassium homeostasis in *B. subtilis* under conditions of extreme potassium limitation. Importantly, we demonstrate that the function of a metal ion can partially be taken over by amino acids.

## MATERIALS AND METHODS

### *B. subtilis* strains and growth conditions.

All *B. subtilis* strains used in this work are derived from the wild-type laboratory strain 168. The *B. subtilis* strains used are listed in Table S1 in the supplemental material. *B. subtilis* was grown in LB medium (34) and in MSSM minimal medium containing glucose and ammonium as the basic sources of carbon and nitrogen, respectively (10). The medium was supplemented with auxotrophic requirements (at 50 mg/liter) as indicated. Plates were prepared by the addition of 17 g Bacto agar/liter (Difco) to the liquid medium.

10.1128/mBio.00861-17.1 TABLE S1  Bacterial strains used in this study. Download TABLE S1, DOCX file, 0.02 MB. Copyright © 2017 Gundlach et al. 2017 Gundlach et al. This content is distributed under the terms of the Creative Commons Attribution 4.0 International license.

### DNA manipulation and transformation.

*E. coli* DH5α (34) was used for cloning experiments. Transformation of *E. coli* and plasmid DNA extraction were performed by standard procedures (34). Restriction enzymes, T4 DNA ligase, and DNA polymerases were used as recommended by the manufacturers. DNA fragments were purified from agarose gels using the QIAquick PCR purification kit (Qiagen, Germany). *Phusion* DNA polymerase was used for the PCR as recommended by the manufacturer. All primer sequences are provided in the supplemental material (Table S2). DNA sequences were determined by using the dideoxy chain termination method (34). All plasmid inserts derived from PCR products were verified by DNA sequencing. Chromosomal DNA from *B. subtilis* was isolated using the peqGOLD bacterial DNA kit (Peqlab, Erlangen, Germany).

10.1128/mBio.00861-17.2 TABLE S2  Oligonucleotides used in this study. Download TABLE S2, DOCX file, 0.01 MB. Copyright © 2017 Gundlach et al. 2017 Gundlach et al. This content is distributed under the terms of the Creative Commons Attribution 4.0 International license.

### Transformation and phenotypic analysis.

Standard procedures were used to transform *E. coli* (34), and transformants were selected on LB plates containing ampicillin (100 µg/ml). *B. subtilis* was transformed with plasmid or chromosomal DNA according to the two-step protocol described previously (35). Transformants were selected on SP plates containing chloramphenicol (Cm) (5 µg/ml), kanamycin (Km) (10 µg/ml), spectinomycin (Spc) (150 µg/ml), tetracycline (Tet) (12.5 µg/ml), or erythromycin (Em) (2 µg/ml) plus lincomycin (Lin) (25 µg/ml).

 Amylase activity in *B. subtilis* was detected after growth on plates containing nutrient broth (7.5 g/liter), 17 g Bacto agar/liter (Difco), and 5 g hydrolyzed starch/liter (Connaught). Starch degradation was detected by sublimating iodine onto the plates.

Quantitative studies of *lacZ* expression in *B. subtilis* were performed as follows. Cells were grown in MSSM medium supplemented with KCl at different concentrations as indicated. Cells were harvested at an OD_600_ of 0.5 to 0.8. β-Galactosidase specific activities were determined with cell extracts obtained by lysozyme treatment as described previously (35). One unit of β-galactosidase is defined as the amount of enzyme that produces 1 nmol of *o*-nitrophenol per min at 28°C.

To assay growth of *B. subtilis* mutants at different potassium concentrations, a drop dilution assay was performed. Briefly, precultures in MSSM medium at the indicated potassium concentration were washed three times, resuspended to an OD_600_ of 1.0 in MSSM basal salts solution. Dilution series were then pipetted onto MSSM plates with 0.5 or 50 mM KCl.

### Plasmids.

Plasmid pAC6 (36) was used to construct a transcriptional fusion of the *ktrAB* promoter region with the *lacZ* gene. For the construction of plasmid pGP2945 containing a *ktrA*-*lacZ* fusion, the region upstream of *ktrA* was amplified using the oligonucleotides JN467/JN371. The PCR product was digested with EcoRI and BamHI PCR and cloned into pAC6 linearized with the same enzymes. Plasmids for the mutant promoter regions were constructed accordingly. All plasmids used in this study are listed in Table S3.

10.1128/mBio.00861-17.3 TABLE S3  Plasmids used in this study. Download TABLE S3, DOCX file, 0.01 MB. Copyright © 2017 Gundlach et al. 2017 Gundlach et al. This content is distributed under the terms of the Creative Commons Attribution 4.0 International license.

### Construction of deletion strains.

Deletion of the *yugO* and *odhA* genes was achieved by transformation with PCR products constructed using oligonucleotides (Table S2) to amplify DNA fragments flanking the target genes and intervening antibiotic resistance cassettes as described previously (37, 38).

### Mapping suppressors by whole-genome sequencing and PCR analysis.

Chromosomal DNA from *B. subtilis* was isolated using the peqGOLD bacterial DNA kit (Peqlab, Erlangen, Germany). To identify the mutations in the mutant strains GP2170, GP2237, GP2238, GP2239, GP2272, and GP2702, the genomic DNA was subjected to whole-genome sequencing. The reads were mapped on the reference genome of *B. subtilis* 168 (GenBank accession number NC_000964) (39). Mapping of the reads was performed using the Geneious software package (Biomatters Ltd., New Zealand) (40). Single nucleotide polymorphisms (SNPs) were considered significant when the total coverage depth exceeded 25 reads with a variant frequency of ≥90%. All identified mutations were verified by PCR amplification and Sanger sequencing.

### Western blotting.

To facilitate the analysis of KtrAB production in *B. subtilis* by Western blot analysis, a triple FLAG tag was fused to the C terminus of KtrB. For this purpose, plasmid pGP2943 and *B. subtilis* strains GP2277, GP2278, and GP2280 were constructed using the vector pGP1331 (41) as outlined in Table S1. For Western blot analysis, *B. subtilis* cell extracts were separated on 12.5% SDS-polyacrylamide gels. After electrophoresis, the proteins were transferred to a polyvinylidene difluoride membrane (PVDF) (Bio-Rad) by electroblotting. Proteins were detected using specific antibodies raised against *B. subtilis* HPr (42) or antibodies recognizing the FLAG tag (Sigma). The primary antibodies were visualized by using alkaline phosphatase (AP)-labeled anti-rabbit IgG secondary antibodies (Promega) and the CDP* detection system (Roche Diagnostics) (43).

### Metabolite analysis.

Metabolites were measured as described previously (44). Briefly, an Agilent 1290 Infinity II ultrahigh performance liquid chromatographic (UHPLC) system (Agilent Technologies) was used for liquid chromatography (LC). The column was an Acquity BEH (ethylene bridged hybrid) amide column (30 by 2.1 mm with 1.7-µm particle size) (Waters GmbH). The temperature of the column oven was 30°C, and the injection volume was 3 µl. LC solvent A was water with 10 mM ammonium formate and 0.1% formic acid (vol/vol), and LC solvent B was acetonitrile with 0.1% formic acid (vol/vol). The gradient was 90% LC solvent B at 0 min, 40% LC solvent B at 1.3 min, 40% LC solvent B at 1.5 min, 90% LC solvent B at 1.7 min, and 90% LC solvent B at 2 min. The flow rate was 0.4 ml min^−1^. An Agilent 6495 triple quadrupole mass spectrometer (Agilent Technologies) was used for mass spectrometry. The source gas temperature was set at 200°C, with 14 liters min^−1^ drying gas and a nebulizer pressure of 24 lb/in^2^. The sheath gas temperature was set at 300°C, and flow was set at 11 liters min^−1^. Electrospray nozzle and capillary voltages were set at 500 and 2,500 V, respectively. Isotope ratio mass spectrometry with ^13^C internal standard was used to obtain absolute quantitative data. Ornithine was calibrated with external standards due to low concentrations in the ^13^C internal standard.

### Determination of cellular potassium pools.

The cellular potassium pools were determined as described previously (19). Briefly, *B. subtilis* cells were cultivated in MSSM medium with potassium and ornithine supplementation as indicated. Cells were pelleted, transferred onto ash-free filter disks, and dried. The dried filter disks were cut into small pieces and reduced to a fluid state through pressure and 2 ml of 65% HNO_3_ for 7 h at 185°C in 25-ml Teflon beakers (PDS-6 pressure digestion system, Loftfields, Göttingen, Germany). After digestion, the fluid content was transferred into an Erlenmeyer flask and diluted with demineralized water to a volume of 50 ml. The total potassium content of the cells in this solution was determined by ICP-OES (inductively coupled plasma optical emission spectrometry) analysis (Optima 5300 DV; PerkinElmer, Waltham, MA, USA). Light emission at 766.49 nm that is indicative of the potassium concentration in the sample was recorded.
