# Supplementary material for: Adaptation of Bacillus subtilis to Life at Extreme Potassium Limitation
Source: mBio. 2017 Jul 5;8(4):e00861-17. doi: 10.1128/mBio.00861-17 (PMC5573677; doi:10.1128/mBio.00861-17)
Supplement: TABLE S1 [file mbo003173372st1.docx]

| **Strain** | **Genotype** | **Reference/ Construction** | **Remarks** |
| --- | --- | --- | --- |
| 168 | *trpC2* | Laboratory collection |  |
| BKE24250 | *∆ahrC::ermC* | 1 |  |
| GP92 | *trpC2 ∆ktrAB::aphA3* | 2 |  |
| GP93 | *trpC2 ∆kimA::cat* | 2 |  |
| GP2079 | *trpC2 ∆ktrC::tet* | 2 |  |
| GP2098 | *trpC2 ∆yugO*::*ermC* | LFH → 168 |  |
| GP2165 | *trpC2 ∆ktrAB::aphA3 ∆kimA::cat* | 2 |  |
| GP2166 | *trpC2 ∆kimA::cat ∆ktrC::tet* | GP2079 → GP93 |  |
| GP2170 | *trpC2 ∆ktrAB*::*aphA3 ∆yugO*::*ermC ∆yobH-uvrX* | GP2098 → GP92 | Spontaneous 200 kb deletion from *yobH* to *uvrX* |
| GP2183 | *trpC2 ∆odhA::ermC* | LFH → 168 |  |
| GP2185 | *trpC2 ∆ahrC::ermC* | BKE24250 → 168 |  |
| GP2187 | *trpC2 ∆ktrAB::aphA3 ∆kimA::cat ∆ahrC::ermC* | GP2185 → GP2165 |  |
| GP2188 | *trpC2* ∆*ktrAB*::*aphA3* ∆*kimA::cat* ∆*odhA::ermC* | GP2183 → GP2165 |  |
| GP2237 | *trpC2 ∆ktrAB::aphA3 ∆kimA::cat* p*_argC_* _C →_ _T operator site_ | Suppressor isolation  of GP2165 | p*_argC_*: TTGAATTAATTTTT-ATT**T**ATGTTATAAT |
| GP2238 | *trpC2 ∆ktrAB*::*aphA3 ∆kimA*::*cat* p*_argC_* _T insertion operator site_ | Suppressor isolation  of GP2165 | p*_argC_*: TTGAATTAATTTTT**T**ATTCATGTTATAAT |
| GP2239 | *trpC2 ∆ktrAB::aphA3 ∆kimA::cat ahrC* _Q22R_ | Suppressor isolation  of GP2165 |  |
| GP2272 | *trpC2* p*_ktrA_*_-S1_ | Suppressor isolation of 168 | p*_ktrA_*: TTGACAAACACTAGAAACAGGAGTAAA**A**T (S1) |
| GP2273 | *trpC2* p*_ktrA_*_-S2_ | Suppressor isolation of 168 | p*_ktrA_*: TTGACAAACACTAGAAACAGGAGTAAATTCTT**A**AGT (S2) |
| GP2274 | *trpC2* p*_ktrA_*_-S3_ | Suppressor isolation of 168 | p*_ktrA_*: TTGACAAACACTAGAAACAGGAGTAAA**C**T (S3) |
| GP2277 | *trpC2 ktrB*-FLAG *spc* | pGP2943 → 168 |  |
| GP2278 | *trpC2* p*_ktrA_*_-S1_ *ktrB*-FLAG *spc* | pGP2943 → GP2272 |  |
| GP2280 | *trpC2* p*_ktrA_*_-S3_ *ktrB*-FLAG *spc* | pGP2943 → GP2274 |  |
| GP2299 | *trpC2 amyE::(*p*_ktrA_-lacZ cat)* | pGP2945 → 168 |  |
| GP2300 | *trpC2 amyE*::(p*_ktrA_*_-S1_-*lacZ cat*) | pGP2946 → 168 |  |
| GP2701 | *trpC2 amyE*::(p*_ktrA_*_-S3_-*lacZ cat*) | pGP2947 → 168 |  |
| GP2702 | *trpC2 ∆ktrC*::*tet ∆kimA*::*cat odhA* _E218STOP_ | Suppressor isolation of GP2166 |  |
| GP2703 | *trpC2 ∆ktrAB*::*aphA3 ∆kimA*::*cat odhA* _RA336EP_ | Suppressor isolation of GP2165 |  |
| GP2714 | *trpC2 amyE*::(p*_ktrA_*_-S2_-*lacZ cat*) | pGP2948 → 168 |  |

Table S1. *B. subtilis* strains used in this study. Arrows indicate construction by transformation. LFH, long flanking homology PCR

References

1. Koo BM, Kritikos G, Farelli JD, Todor H, Tong K, Kimsey H, Wapinski I, Galardini M, Cabal A, Peters JM, Hachmann AB, Rudner DZ, Allen KN, Typas A, Gross CA. 2017. Construction and analysis of two genome-scale deletion libraries for *Bacillus subtilis*. Cell Syst. 4: 291-305.
2. Gundlach J, Herzberg C, Kaever V, Gunka K, Hoffmann T, Weiß M, Gibhardt J, Thürmer A, Hertel D, Daniel R, Bremer E, Commichau FM, Stülke J. 2017. Control of potassium homeostasis is an essential function of the second messenger cyclic di-AMP in *Bacillus subtilis*. Sci Signal 10: eaal3011.
