# Supplementary material for: Adaptation of Bacillus subtilis to Life at Extreme Potassium Limitation
Source: mBio. 2017 Jul 5;8(4):e00861-17. doi: 10.1128/mBio.00861-17 (PMC5573677; doi:10.1128/mBio.00861-17)
Supplement: TABLE S2 [file mbo003173372st2.docx]

| **Oligo** | **Sequence** | **Gene** |  |
| --- | --- | --- | --- |
| CZ144 | **CCTATCACCTCAAATGGTTCGCTG**GTAAAAATCTTCCCAATTCATTC | LFH_*odhA*_rev_up, **kan^R^** |  |
| CZ145 | GGGTATGGCTGACGGCAAAG | LFH_*odhA*_fwd_up |  |
| CZ146 | GGCTCTGTTCAAGGATCAGC | LFH_*odhA*_seq_fwd |  |
| CZ147 | **CGAGCGCCTACGAGGAATTTGTATCG**GAACGTATTGTATCTGATAGCTT | LFH_*odhA*_fwd_down, **kan^R^** |  |
| CZ148 | GCCCAGTATACCGACTTGCG | LFH_*odhA*_rev_down |  |
| CZ149 | CATAAGATAAAGCGATATACATC | LFH_*odhA*_seq_rev |  |
| JN271 | **CCTATCACCTCAAATGGTTCGCTG**CGGATAAATAACGGCCACCTGA | LFH_*yugO*_rev_up, **kan^R^** |  |
| JN272 | GATATGGCGGAAATTGAGCGG | LFH_*yugO*_fwd_up |  |
| JN273 | **CCGAGCGCCTACGAGGAATTTGTATCG**CATACAAGGTGCTCGAAACAGATC | LFH_*yugO*_fwd_down, **kan^R^** |  |
| JN274 | GCGTATAAAGTCAATATGTTTGCGTTAC | LFH_*yugO*_rev_down |  |
| JN275 | GGAAGATTTTATGTCTCAGCACAAG | LFH_*yugO*_fwd_seq |  |
| JN276 | CCTTGTATATTTCTTCGAAAAAGCCTG | LFH_*yugO*_rev_seq |  |
| JN371 | TTTGGATCCCCAATCGTTGGTTCCCCCG | rev_*ktrA*, control region (-RS) | BamHI |
| JN467 | AAAGAATTCGCGTTTTACCCTCCCCTTTTTC | fwd_*ktrA*, control region | EcoRI |
| JN716 | AAAGGATCCGTCTTTATTTTAGAATACAGCAACCCCG | fwd_*ktrB* in pGP1331 | BamHI |
| JN717 | TTTGTCGACCCCTGTAAACACTTCGCCATCA | rev_*ktrB* in pGP1331 | SalI |

Table S2. Oligonucleotides used in this study
