# Supplementary material for: Adaptation of Bacillus subtilis to Life at Extreme Potassium Limitation
Source: mBio. 2017 Jul 5;8(4):e00861-17. doi: 10.1128/mBio.00861-17 (PMC5573677; doi:10.1128/mBio.00861-17)
Supplement: TABLE S3 [file mbo003173372st3.docx]

| **Plasmid** | **Description** | **Construction/ Reference** |  |
| --- | --- | --- | --- |
| pAC6 | Vector for the construction of transcriptional *lacZ* fusions that can be integrated at the *amyE* site. | 1 |  |
| pGP1331 | Integrative vector for the fusion of 3x FLAG tag to the C-terminus of a protein, keeping expression under the natural promoter. | 2 |  |
| pGP2943 | pGP1331/ BamHI+SalI | PCR Prod. *ktrB* JN716/717/ BamHI+SalI |  |
| pGP2945 | pAC6/ EcoRI+BamHI | PCR Prod. p*_ktrA_* JN467/JN371/ EcoRI/BamHI |  |
| pGP2946 | pAC6/ EcoRI+BamHI | PCR Prod. p*_ktrA_*_-S1_ JN467/JN371/ EcoRI/BamHI | amplified using GP2272 as template |
| pGP2947 | pAC6/ EcoRI+BamHI | PCR Prod. p*_ktrA_*_-S3_ JN467/JN371/ EcoRI/BamHI | amplified using GP2274 as template |
| pGP2948 | pAC6/ EcoRI+BamHI | PCR Prod. p*_ktrA_*_-S2_ JN467/JN371/ EcoRI/BamHI | amplified using GP2273 as template |

Table S3. Plasmids used in this study

References

1. Stülke J, Martin-Verstraete I, Zagorec M, Rose M, Klier A, Rapoport G. 1997. Induction of the *Bacillus subtilis* *ptsGHI* operon by glucose is controlled by a novel antiterminator, GlcT. Mol Microbiol 25: 65-78.
2. Lehnik-Habrink M, Pförtner H, Rempeters L, Pietack N, Herzberg C, Stülke J. 2010. The RNA degradosome in *Bacillus subtilis*: identification of CshA as the major RNA helicase in the multi-protein complex. Mol Microbiol 77: 958-971.
